# Simultaneous Bilateral Rupture of the Patellar Tendon and Medial Collateral Ligament: A Case Report and Literature Review

**DOI:** 10.1155/2020/8862600

**Published:** 2020-10-13

**Authors:** Jean G. Louka, Damien Pourre

**Affiliations:** Department of Orthopaedic Surgery and Traumatology, Simone Veil Hospital, Eaubonne, France

## Abstract

Bilateral rupture of the patellar tendon is considered an uncommon and rare musculoskeletal injury. The association of this lesion with medial collateral ligament tear appears to be exceedingly rare. We present the case of a combined rupture of the medial collateral ligament (MCL) and the patellar tendon of both knees in a 48-year-old man, after falling 2 meters down an embankment. While there are numerous publications concerning associated MCL tears and other knee ligaments, a combination of MCL tear with a patellar tendon rupture is very rare. In addition, our case presents the first case recorded in the literature, involving both knees of a patient. The clinical case is described and discussed following a review of the literature. The symmetrical knee injury was treated with a primary direct repair of the MCL tears and using a suture anchor fixation of the patellar tendon ruptures, which was reinforced by a stainless steel wire and an autograft of the ipsilateral quadriceps tendon.

## 1. Introduction

Bilateral rupture of the patellar tendon is considered an uncommon and rare musculoskeletal injury [1]. The association of this lesion with the medial collateral ligament (MCL) tear appears to be exceedingly rare [2]. The authors describe the case of a 48-year-old man with a bilateral rupture of the patellar tendons, combined with a bilateral complete tear of the MCL. These lesions occur frequently in isolation [3, 4], but the uniqueness of this case lies in the bilateral combination of both lesions. No reports of an exactly similar case have been found in the literature.

In 2014, De Baere et al. [2] described the first case of a unilateral, combined MCL, and patellar tendon lesion. The lesions described were treated with the transosseous repair of the patellar tendon along with fascia lata allograft augment, and medial collateral ligament direct suture reinforced with semitendinosus tendon autograft. They reported a good functional result.

The purpose of this case report is to highlight a very rare entity by describing both the clinical and radiological findings in addition to the surgical technique used. We also illustrate the postoperative rehabilitation. The patient was informed that the case data would be submitted for publication and agreed upon.

## 2. Case Report

We present the case of a 48-year-old man, complaining of a bilateral knee injury and functional disability. The patient fell about two meters down an embankment one hour before the presentation and was unable to stand up due to pain, so he was brought to our hospital by ambulance.

Clinical examination revealed a marked bilateral swelling of both knees, severe pain at the passive mobilization of the knee joints, pain-limiting active flexion to less than 30°, and an inability to actively extend the knee joints or to perform an active straight leg raise bilaterally. Additionally, weight-bearing had hardly been possible. There was a loss of fullness and a palpable deficit at the inferior poles of his patellae. Testing of the ligamentous knee joint stabilizers was significantly limited by guarding due to the severe sharp pain. The patient's medical history revealed that a couple of years earlier, he had a right knee sprain resulting in an acute rupture of the anterior cruciate ligament (ACL), which was treated conservatively. The patient does not take any drugs regularly, and we note that he is allergic to levofloxacin. The weight and height of the patient were recorded at the physical examination as 107 kilograms and 180 cm, with a body mass index (BMI) of 33.

**Figure 1:**
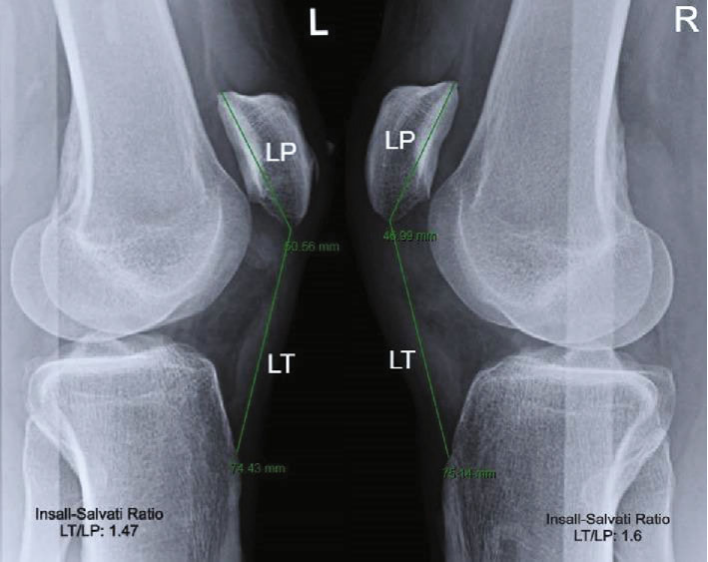
Lateral knee radiographs after bilateral patellar tendon rupture, showing patella alta, knee effusions, and irregularity of the patellar tendons. Confirmed diagnosis using the Insall-Salvati ratio.

Plain radiographs of his knees showed bilateral knee effusions with patella alta (high-riding patellae) on both anteroposterior and lateral views. Insall-Salvati ratios measured 1.6 and 1.47 for right and left knee, respectively (Figure 1) (normal values range from 0.8 to 1.2; patella alta > 1.2 and patella baja < 0.8). Moreover, the irregularity and incongruity of the patellar tendons on the lateral radiographs were additional signs suggestive of the extensor mechanism's rupture and consistent with the tendons' rupture from the lower pole of the patellae bilaterally.

Our patient was operated under general anesthesia 48 hours after the accident. He was placed in the supine position. The clinical examination under anesthesia of both knees demonstrated a full instability of the MCL with a valgus stress test at both 0 and 30° of knee flexion. No laxity was demonstrated in the remaining ligaments of the knee joint. The lower extremities were prepared and draped together in the usual sterile fashion. The intervention was performed without the use of a tourniquet.

**Figure 2:**
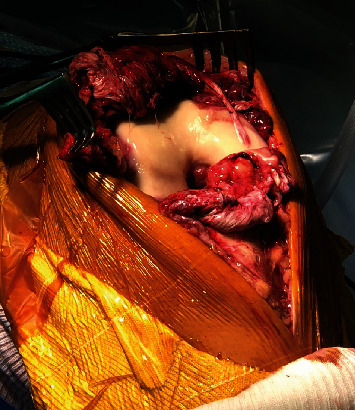
Intraoperative photo showing the ruptured patellar tendon of the right knee.

Our ligamentous reconstruction was approached by an anterior longitudinal midline incision. Dissection was carried down through the skin and subcutaneous tissues to the level of the patellar and quadriceps paratenon, which were carefully preserved. The patellar tendon rupture was identified near the proximal osteotendinous junction bilaterally (Figure 2). The hemarthrosis was evacuated, and the joint was copiously irrigated. A chronic tear of the right ACL was identified, with the proximal portion of the ligament missing and only scar tissue remaining with the ACL stump adhering to an intact Posterior Cruciate Ligament (PCL). A complete proximal (femoral) MCL tear was identified bilaterally, confirming our clinical suspicion. The medial and lateral retinacula, which were involved as well, were identified for later repair.

At first, with the same incision, the MCL tear was approached. Proximal reinsertion at the level of the medial femoral condyle using a DePuy Mitek super QuickAnchor™ Plus DS® was done to ensure the stability of the medial motion plane. Then, after debridement of the tendinous tissue and visualization of the inferior pole of the patella, three DePuy Mitek super QuickAnchor™ Plus DS® were screwed into the medial, middle, and lateral thirds of the patella in the proper coronal plane. The purchase of the anchors was tested as we were able to deliver the patella to the distal extent of the incision by pulling on the anchor sutures. Then, a circumferential 1.2 mm thick stainless steel wire was passed through the center of the thickness of the patella superiorly and the tibial tuberosity inferiorly. Gradual tension was applied on the metallic wire to obtain the optimal patellar height, confirmed by an intraoperative lateral X-ray. One suture in each anchor was used to create a running Krackow stitch distally through the tendon, ensuring that full-thickness bites were obtained.

The second limb of each suture was passed in a locked fashion through the proximal free tendon and tied within the substance of the tendon. The additional suture within each anchor was incorporated into the repair for reinforcement in a simple continuous fashion. The reconstruction was further protected by a strip of quadriceps tendon measuring 10 × 1 cm, long enough to cover the patellar tendon, which was harvested and turned down. The edges of the turned down quadriceps tendon were fixed to the underlying patellar tendon using slowly absorbable interrupted sutures (Vicryl 2.0). Next, the medial and lateral retinacula tears were repaired using interrupted No. 2 PDS sutures (Ethicon; Somerville, New Jersey, USA). The strength of the repair was tested bilaterally through a gentle range of motion; a flexion up to 130° was possible.

**Figure 3:**
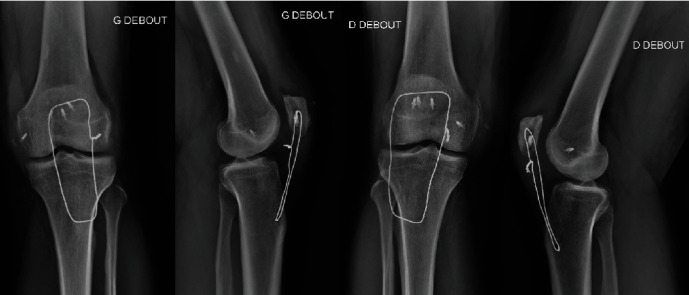
Radiographic control 10 weeks after surgery.

Postoperatively, the legs were placed in knee immobilizer braces, with the knees locked in full extension. The postoperative course was uneventful, and radiographic control was satisfactory (Figure 3). On the second postoperative day, the patient began ambulation with a walker while keeping the extension knee braces. Full weight-bearing was permitted as tolerated, along with isometric quadriceps-strengthening exercises. The rest of the protocol is as follows: knee flexion exercises limited to 45° were started at the second postoperative week. He had no pain and reached 45° of active bilateral knee flexion. He had an active flexion of 80° at the sixth week, and the knee braces were discontinued. In the eighth postoperative week, the patient achieved a bilateral active complete knee extension and could walk without crutches. As part of his daily physiotherapy program, he was allowed full knee flexion along with a focus on muscle strengthening exercises. Twelve weeks after surgery, the patient presented 100° maximum bilateral knee flexion and returned to work.

**Figure 4:**
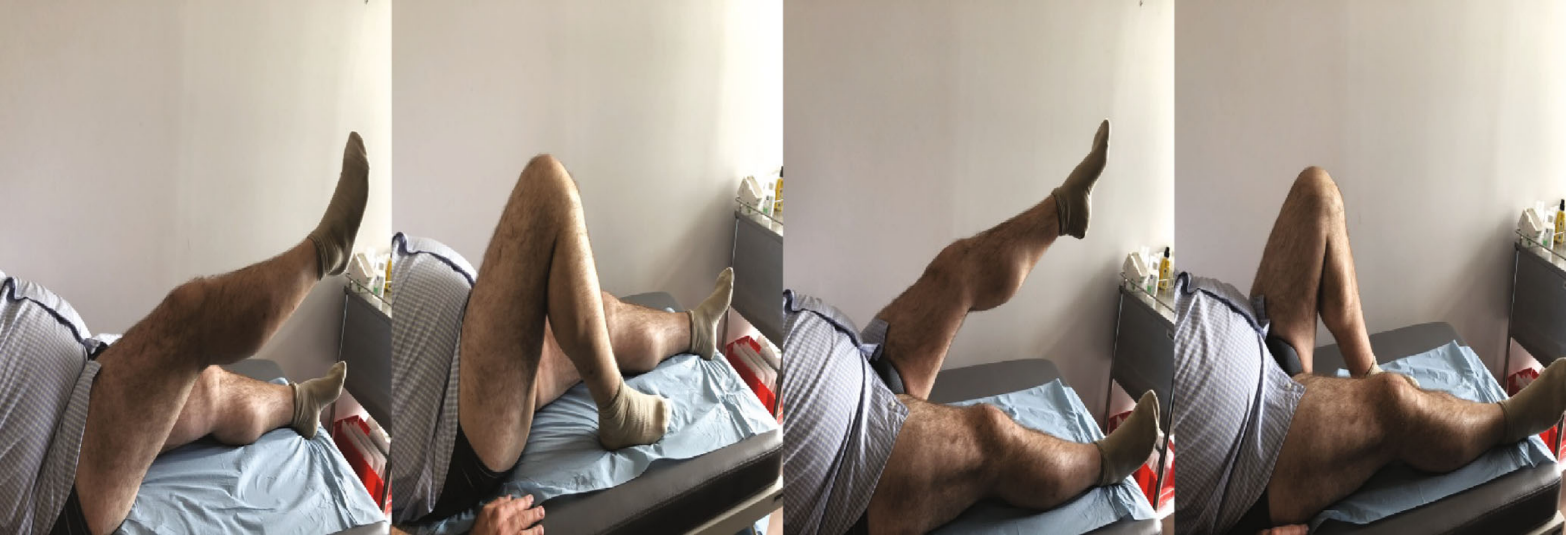
Seven months after surgery: 135° flexion, 0° extension at both knees.

Upon examination seven months after surgery, the patient showed an adequate range of motion of both knees (135° flexion, 0° extension) (Figure 4). Quadriceps muscle, the primary contributor to knee joint stability, had a good strength with no clinical signs of muscular atrophy or extensor lag. The patient denied any sense of instability, and, consequently, he returned to his recreational sports activities. In addition, he reported feeling that his knees were as strong as they were before the accident. At the final follow-up 12 months after the injury, the patient was symptom-free and extremely satisfied as he recovered completely.

## 3. Discussion

To the authors' best knowledge, this is the first recorded case of a bilateral rupture of the patellar tendon coupled with the MCL complete bilateral tear. The literature contains no descriptions of similar cases. Conversely, combined injury of the MCL with other knee structures, especially the ACL, is well known, making it the most common multiligament knee injury [5].

At the start, when approaching the subject of knee injuries, where ligament injuries account for up to 40% of all cases, medial collateral ligament rupture should be strongly considered as the MCL is the most commonly injured ligament in the knee [6].

According to the American Medical Association's recommendation, sprains of the knee collateral ligaments are graded into three groups based on their severity [7]: grade I implies either a stretch injury or microtears with no instability; grade II implies a partial MCL tear with mild to moderate joint laxity; grade III is a complete tear of the ligament with gross instability. Additionally, MCL tears occur as isolated lesions or often in combination with injuries to other ligamentous structures such as the ACL and menisci. Fetto and Marshall found an 80% incidence of combined ligament injury with grade III MCL tears [8]. The extrasynovial location of the MCL combined with its abundant vascular supply offers an excellent healing potential [9, 10]. Partial MCL tears (grades I and II) usually heal well with nonoperative treatment, whereas it is debatable whether grade III injury treatment should be nonoperative or surgical [11, 12]. In general, femoral-based grade III tears can be treated conservatively. Contrarily, when combined with other knee ligaments where anteroposterior, valgus, and rotatory instability can develop, there is a propensity to treat them surgically [13–16].

Kovachevich et al. [17] reviewed the treatment of MCL tears in the combined ligament injured knee. They concluded that a complete tear of the MCL has a poor healing capacity when left on his own in these severely traumatized knees and surgical treatment is indicated. Good results can be obtained with either direct repair of the MCL or reconstruction with semitendinosus autograft or synthetic graft.

Besides, the rupture of the patellar tendon is a rare injury, with an incidence rate of less than 0.5 percent of the United States population per year [18] and a peak incidence in men during their third and fourth decades [19]. To explain the rupture of the patellar tendon, one must first tackle the knee's extensor mechanism. The extensor mechanism comprises the quadriceps muscle and tendon, the patella, the patellar tendon, the patellar retinacula, and the tibial tubercle. Furthermore, the patellar tendon rupture is the third most common cause of extensor mechanism dysfunction after patellar fracture and quadriceps tendon rupture [4]. The vast majority of those injuries are unilateral [20, 21]. Only a few rare cases of bilateral patellar tendon rupture have been reported. Typically, bilateral tendon injury has been associated with systemic diseases such as renal failure, diabetes mellitus, rheumatoid arthritis, systemic lupus erythematosus, or a combination of those diseases [22–33]. Furthermore, several authors reported a correlation between tendon rupture and the use of steroids and fluoroquinolones [34–38]. All the aforementioned factors are accused of weakening collagenous structures and the structural integrity of the tendons, leading to their rupture.

Generally speaking, bilateral ruptures may be missed on initial presentations in up to 50% of cases due to lack of an uninjured contralateral side for reference [28, 39, 40]. Accordingly, bilateral patellar tendon rupture is a challenging diagnosis, frequently missed on clinical examination. In order to avoid missing this rare entity, some authors suggest that the position of each patella be determined by the lateral radiographic assessment of the Insall-Salvati ratio [19]. The best way to measure this ratio is through a lateral view of the knee in slight flexion (ideally, 30°) [41]. The Insall-Salvati ratio is calculated by dividing the length of the patellar tendon (LT) measured from the inferior pole of the patella to the tibial tuberosity where it is inserted, over the greatest diagonal length of the patella (LP) as measured from the upper pole to the lower nonarticular pole of the patella [41, 42]. An LT/LP ratio greater than 1.2 indicates a patella alta [41–44], which helps diagnose a possible patellar tendon rupture, particularly in acute settings. However, bilateral patella alta differential diagnosis includes patellar subluxation, recurrent dislocation of the patella, and chondromalacia patellae [44–46].

The use of ultrasound and MRI (magnetic resonance imaging) may play a role as an adjunct to standard imaging in the evaluation of such unusual cases. MRI is the most sensitive imaging modality for the diagnosis of tendon rupture; yet, it is more costly and less available [47]. In addition, these imaging modalities help in the diagnosis and characterization of the surrounding soft tissue injuries where other tendons, ligaments, or menisci may be involved.

The typical mechanism of rupture of the patellar tendon is the result of an unanticipated flexion of the knee arising around the same moment as quadriceps contraction [48, 49]. Huberti et al. [50] described the concept of the extensor mechanism force ratio, which relates the degree of knee flexion to the probability of tendon ruptures. This ratio is the fraction of the force found in the patellar tendon divided by the quadriceps tendon. It is greater than 1.0 with a knee flexion less than 45°, putting the quadriceps tendon at a higher risk of injury.

Contrarily, with the knee joint flexion greater than 45°, the patellar tendon is at a higher risk of failure. Zernicke et al. [51] found that a healthy human patellar tendon needs a force of 17.5 times the weight of body in a young individual to rupture. In order to bring this into context, it is estimated that ascending stairs can produce a force of 3.3 times the body weight within the patellar tendon.

As with unilateral patellar tendon ruptures, bilateral ruptures can be either spontaneous or posttraumatic depending on the causal mechanism. Several hypotheses have been proposed as it can be difficult to determine whether the tendon was actually been exposed to sufficient stress that caused a traumatic rupture. Conversely, it may be difficult, even in the presence of a predisposing factor, to determine its level of contribution to the so-called “spontaneous” patellar tendon rupture. Our patient did not have any personal or family medical history; however, he was obese with a BMI of 33. In obese patients, it could be hypothesized that excess weight put a constant excessive local stress on the load-bearing tendon, which causes overuse tendinopathy and leads to the rupture of the tendon. In addition, it has also been reported that the fatty degeneration of the tendon microstructure observed in obese patients could predispose it to rupture [52–54]. Interestingly, obesity shares a common pathogenetic pathway with diabetes mellitus, where an increase in advanced glycation end products has detrimental effects on the tendons [55].

Various authors have tried to classify patellar tendon ruptures on the basis of the location (distal patellar pole, tendon midsubstance, or tibial tubercle), configuration (transverse, *Z*-type, and inverted-U based), and duration between injury and repair (acute < 2 weeks vs. chronic > 2 weeks) [56–60]. The Siwek and Rao classification, dividing the patellar tendon ruptures as acute or chronic, appears to be the main perioperative factor that correlated with postoperative outcomes. Interestingly, the rupture site of the patellar tendons in almost all bilateral cases was symmetrical in localization [19]. Furthermore, in certain cases, the rupture of the bilateral patellar tendons is described as quasisimultaneous, indicating that the rupture of the tendon in one knee placed extreme stress on the opposite knee, resulting in symmetrical tendon rupture during the same event (e.g., jumping/landing and falling down/standing up) [19, 40, 61–63].

According to a study by Clayton and Court-Brown [5], patellar tendon rupture involved only 0.6 percent of 2,794 soft tissue injuries. Much rarer is a simultaneous rupture of both the ACL and the ipsilateral patellar tendon [64]. Moreover, a limited number of cases reported a simultaneous rupture of the ACL, the MCL, and the patellar tendon [64]. Futch et al. [65] reported a case of a combined ACL and patellar tendon rupture in a young athlete. This knee was treated with a reconstruction of the ACL and a suture of the patellar tendon, which was reinforced with a patellar tendon allograft.

## 4. Conclusion

In the case we presented, the combination of a grade III MCL tear with a patellar tendon rupture is absolutely not to be considered as a multiligament injured knee. During the surgical intervention required for the patellar tendon rupture, obvious clinical valgus instability justified surgical treatment of the MCL tear.

In front of the unusual presentation, especially where the physical examination is impossible, it is advisable to routinely order an MRI of the knee, to assess the extent of injury and to look for any associated injuries. Moreover, our patient suffered from bilateral patellar tendons and MCL rupture. In order to protect the tendon sutures and to ensure early mobilization and faster recovery, we decided to use the quadriceps tendon autografts and the stainless steel wires, as a double protection, to secure solid stability of the mounting, in this difficult-to-treat unusual case.
